# Transgenic rice *Oryza glaberrima* with higher CPD photolyase activity alleviates UVB-caused growth inhibition

**DOI:** 10.1080/21645698.2021.1977068

**Published:** 2021-12-22

**Authors:** Gideon Sadikiel Mmbando, Mika Teranishi, Jun Hidema

**Affiliations:** Graduate School of Life Sciences, Tohoku University, Sendai, Japan

**Keywords:** African rice, *Agrobacterium*, bioengineering, callus induction, regeneration

## Abstract

The ultraviolet B (UVB) sensitivity of rice cultivated in Asia and Africa varies greatly, with African rice cultivars (*Oryza glaberrima* Steud. and *O. barthii* A. Chev.) being more sensitive to UVB because of their low cyclobutane pyrimidine dimer (CPD) photolyase activity, which is a CPD repair enzyme, relative to Asian rice cultivars (*O. sativa* L.). Hence, the production of UVB-resistant African rice with augmented CPD photolyase activity is of great importance, although difficulty in transforming the African rice cultivars to this end has been reported. Here, we successfully produced overexpressing transgenic African rice with higher CPD photolyase activity by modifying media conditions for callus induction and regeneration using the parental line (PL), UVB-sensitive African rice TOG12380 (*O. glaberrima*). The overexpressing transgenic African rice carried a single copy of the CPD photolyase enzyme, with a 4.4-fold higher level of CPD photolyase transcripts and 2.6-fold higher activity than its PL counterpart. When the plants were grown for 21 days in a growth chamber under visible radiation or with supplementary various UVB radiation, the overexpressing transgenic plants have a significantly increased UVB resistance index compared to PL plants. These results strongly suggest that CPD photolyase remains an essential factor for tolerating UVB radiation stress in African rice. As a result, African rice cultivars with overexpressed CPD photolyase may survive better in tropical areas more prone to UVB radiation stress, including Africa. Collectively, our results provide strong evidence that CPD photolyase is a useful biotechnological tool for reducing UVB-induced growth inhibition in African rice crops of *O. glaberrima*.

## 1. Introduction

Ultraviolet-B (UVB) radiation (280–315 nm) in sunlight reduces photosynthesis and protein synthesis, thereby diminishing plant growth and productivity.^1^ Although the Montreal Protocol has prevented a significant increase in UV-B (280–315 nm) radiation,^2^ we are now observing an unprecedented increase in UVB levels in the Arctic due to stratospheric ozone depletion in 2020.^3^ Therefore, rising UVB levels due to depletion of the stratospheric ozone layer remains a serious global concern.^3,4^ Rice is one of the most important staple grains, grown worldwide in regions with different climates. UVB-sensitive rice cultivars identified in Asian species are cultivated in tropical regions where the amount of UVB radiation is relatively high.^5–8^ For example, two *indica* rice cultivars, Surjamkhi^1^ and Kasalath (*O. sativa* L., ssp. *indica*),^9^ are far more sensitive (i.e. hypersensitive) to UVB-induced growth inhibition than the *japonica* UVB-sensitive rice cultivar (*O. sativa* L.). Recently, we demonstrated that the major African rice cultivars (*O. glaberrima* and *O. barthii*) grown on the African continent with higher UVB radiation are even more sensitive (i.e. super-hypersensitive) to UVB than Surjamkhi.^10^ Therefore, cultivars with low resistance to UVB damage, especially those from tropical countries, including African ones, may be less productive under current environmental conditions, and even prone to extreme injury from increased UVB radiation as a result of ozone layer depletion.^3,4,11^

There are two major UV-induced forms of DNA photodamage: cyclobutane pyrimidine dimers (CPDs) and pyrimidine (6–4) pyrimidone photoproducts [(6–4) photoproducts]. This DNA damage occurs between adjacent pyrimidines of the same strand, of which CPDs constitute approximately 75%, with (6–4) photoproducts accounting for the remaining damage.^12^ Plants have evolved various mechanisms for coping with UVB-induced DNA damage, such as photoreactivation (i.e. photorepair) and nucleotide excision repair (also known as dark repair). Photorepair is the major and most effective mechanism utilized by plants to repair UVB-induced DNA damage^13–15^; it is mediated by photolyase that uses blue/UVA light as its energy source to monomerize or reverse the dimers. We previously demonstrated that UV-sensitive Asian rice cultivars exhibit a significantly weaker ability to repair CPDs via photorepair than UV-resistant Asian cultivars^15,16^ because of a decreased photolyase function that results from spontaneously occurring polymorphisms in the CPD-specific photolyase (CPD photolyase/CPD photoreactivation enzyme) gene.^6–9^ Various CPD photolyase genotypes have been identified in both cultivated and wild rice species, with specific correlations found between the genotypes and activity of the strains analyzed.^7^ However, in our recent work, no correlations were observed between genotypes and activity, indicated by variations in activity, and differences in the UVB resistance index among African rice cultivars of the same genotype arose from differences in CPD photolyase content.^10^ The UVB sensitivity of rice, thus, depends on the total CPD photolyase activity, which is affected by its genotype and the levels in plant cells. In fact, UVB-resistant transgenic rice plants showing increased CPD photolyase activity were generated by using the UVB-resistant *O. sativa japonica* cultivar Sasanishiki as the parental line (PL) plant,^17^ as well as Norin 1 and Surjamkhi.^18^ Therefore, increasing CPD photolyase activity is arguably an effective way to confer greater resistance to UVB in *O. sativa* species. However, UVB-sensitive *O. glaberrima* plants have a different genetic background than *O. sativa*, and the extent to which an increase in CPD photolyase can mitigate UVB-induced growth inhibition remains unclear.

African landraces reportedly carry various interesting traits that could allow for sustainable and less demanding agricultural production.^19^ Moreover, *O. glaberrima* cultivars have a large potential for cultivation in many areas of Africa. Thus, introduction of the UVB-resistant trait into this species using genetic engineering may help to increase its yield, thereby going a long way toward eliminating hunger and poverty. Surprisingly, although *O. glaberrima* is highly sensitive to UVB,^10^ there are no reports on the generation of transgenic rice plants that increase UVB resistance using *O. glaberrima* as a PL plant. This could be attributable to a lack of concern or interest in genetic manipulation, possibly due to the absence of the appropriate *in vitro* regeneration protocols for local cultivars.^20,21^ In fact, difficulties in the transformation and regeneration of African local cultivars, such as *O. glaberrima*, have been reported in several studies.^20–23^

In this study, we successfully generated transgenic rice plants overexpressing CPD photolyase using the UVB-sensitive African rice TOG12380 (*O. glaberrima*) as the PL plant. This transgenic plant was found to have greater resistance to UVB-induced growth inhibition than PL plants. Thus, this study provides insights that are valuable for the development of UVB-tolerant African rice cultivars.

## 2. Materials and Methods

### 2.1. Construction of Vectors and Transformation of Rice

A binary vector, pPZP2Ha3, was used for rice transformation.^24^ This vector contains the hygromycin phosphotransferase (*hpt*) gene as a selection marker under the control of the cauliflower mosaic virus 35S promoter. The cDNA of CPD photolyase, previously cloned from the UV-resistant rice cultivar Sasanishiki (*O. sativa* L. ssp. *japonica*),^25^ was subcloned into pPZP2Ha3 in the sense orientation, and then into the subclones were transferred into the *Agrobacterium* strain EHA101.^26^ Suitable media for callus induction and regeneration were tested using several African rice varieties, namely TOG12380, TOG14928, MB3, C7251, Jiakawo Wodewo (*O. glaberrima*), and TOB7307 (*O. barthii*). The full protocol is detailed in the Supplementary Material. Briefly, calli were induced from mature seeds of the African UVB-sensitive rice cultivar TOG12380 (*O. glaberrima*) on Murashige and Skoog (MS) medium^27^ modified with an increased sucrose concentration of 50 g L^−1^, according Brisibe et al. (1990).^23^ Calli were transformed as described by Kojima et al. (2000).^28^ One line generated from the hygromycin-resistant callus (T_0_ plants) derived from TOG12380 was selected [TOG12380-photoreactivation enzyme overexpressing (OxPHR) line], and the self-fertilized plants (T_1_) of the T_0_ plants were generated. T_1_ plants grown in half-strength MS media containing hygromycin (hygromycin medium) were transferred to soil, and self-fertilized plants (T_2_) were generated from 12 hygromycin-resistant T_1_ plants. Since heterozygous and homozygotes were mixed in the T_1_ plant, T_2_ seeds were obtained from each individual. The germination rate of T_2_ seeds in hygromycin medium was detected, and a 100% germinated line was used as a homozygous line. The resulting T_2_ or self-fertilized T_3_ lines were used in the experiments.

### 2.2. Plant Growth Conditions

Transgenic and parental rice seeds were grown as previously described.^10^ Plants were grown for 21 days under visible radiation, with or without supplementary UVB radiation, in a growth chamber (ESPEC MIC CORP., Osaka, Japan), under a 12-h/12-h photoperiod and corresponding temperatures of 27°C/17°C. Visible light was supplied by a combination of metal halide lamps (MT 400 DL/BUD; Iwasaki Electric Ltd. Co., Saitama, Japan), and high-pressure sodium lamps (NH360DL; Iwasaki Electric Ltd. Co.) positioned atop the chamber, equipped with a heat-absorbing filter (ESPEC MIC Corp.).^29^ Photosynthetically active radiation (PAR) was recorded using a data logger (LI-1000; Li-Cor Inc., Lincoln, NE, USA) and an L1-190SA sensor (Li-Cor Inc.). The PAR was adjusted so that approximately 350 µmol photon m^−2^ s^−1^ reached the top of the plants. UVB radiation was supplied by three UVB bulbs (FL20SE; Toshiba Co., Tokyo, Japan) filtered through a UV29 glass filter (Toshiba Glass Co., Ltd., Shizuoka, Japan). The UVB intensity just above a given plant was 0.4, 0.8, or 1.2 W m^−2^. Plants (n = 9–10) receiving UVB were grown under the same photoperiod as those plants grown with visible radiation. The UVB intensity and spectral distribution were measured using a spectroradiometer (USR-45 DA; Ushio Inc., Tokyo, Japan). Biologically effective UVB radiation (UVB_BE_) was calculated using the plant action spectrum of Caldwell,^30^ which was normalized to unity at 300 nm. When the plants were grown with supplementary UVB radiation at 0.4, 0.8, or 1.2 W m^−2^, the UVB_BE_ was 4.9, 9.8 m or 14.7 kJ m^−2^ day^−1^, respectively.

To evaluate the levels of CPD photolyase gene transcripts and photorepair activity *in vitro*, both transgenic and PL seedlings were grown for 13 days under visible radiation in a growth chamber until the 3rd leaves (the youngest leaves of 13-day-grown seedlings) had completely expanded. The fully expanded 3rd leaves were used in the experiments.

To examine the steady-state CPD levels in the 3rd leaves of transgenic and PL plants, the plants were grown under visible radiation without supplementary UVB radiation in a growth chamber until the 3rd leaves had completely expanded. Then, three-quarters of the potted plants grown under visible radiation were supplemented with UVB radiation at 0.4, 0.8, or 1.2 W m^−2,^ whereas the remaining plants were maintained under visible radiation only.^17^ Over the next 36 h, the 3rd leaves were detached every 12 h, flash frozen in liquid nitrogen, and stored at – 80°C until CPD analysis.

### 2.3. Southern Blot Analysis

Genomic DNA samples were isolated from homozygous T_2_ plants and PL plants using the cetyltrimethylammonium bromide (CTAB) method, as previously described.^7^ The isolated genomic DNA was digested with either *Bam*HI or *Pst*I and subjected to southern blot analysis with a CPD photolyase probe, as previously described.^17^

### 2.4. RNA Isolation and Quantitative Real-time RT-PCR Analysis

Total RNA was extracted from the 3rd leaves using the RNeasy Plant Mini Kit (Qiagen Inc., Valencia, CA, USA). Reverse transcription was performed with an oligo(dT) primer and a random 6-mer mixture using the Prime Script RT Reagent Kit (Takara Bio Inc., Shiga, Japan) according to the manufacturer’s instructions. Quantitative real-time reverse transcription PCR was performed as described in our recent paper.^10^ Actin served as a normalization control.^31^

### 2.5. Analysis of Photolyase Activity in Leaf Extracts

The preparation of protein extracts from rice leaves, and all procedures for the treatment of DNA with UV endonuclease and alkaline agarose gel electrophoresis, have been described in detail by Hidema et al.^16^ Briefly, the fully expanded 3rd leaves were homogenized using a chilled mortar and pestle in a homogenate buffer (80 mM potassium phosphate buffer, pH 7.2, 5 mM EDTA, 2 mM DTT, 0.2 mg ml^−1^ BSA, and 10% glycerol). The homogenate was centrifuged at 20,000 × *g* for 20 min at 4°C, after which the supernatant was desalted by passage through a Bio-Gel P6DG spin column (Bio-Rad, Hercules, CA, USA). The filtrate was used as a soluble protein. Lambda DNA-50 μg ml^−1^ in 0.1 × TE buffer (1 × TE buffer consisting of 10 mM Tris-HCl, pH 8.0, 1 mM EDTA) was irradiated at 10 J m^−2^ of 254 nm radiation (germicidal lamp; Toshiba Co.), resulting in approximately 150 CPD per megabase (Mb) (CPD Mb^−1^), which was used as the substrate. The DNA was diluted with an equal volume of 2× reaction buffer (1× reaction buffer consisting of a 40-mM potassium phosphate buffer, pH 7.2, 5 mM EDTA, 2 mM DTT, 0.2 mg ml^−1^ BSA, and 80 mM NaCl) and then mixed with the soluble protein. The formation of photolyase-CPD complex in a given mixture sample was facilitated by incubation in the dark, for 15 min at 30°C, followed by exposure to continuous blue light (four blue fluorescent tubes [20B-F; Toshiba Co.]) at a 20-cm distance for 0.5 h to 2 h. All manipulations were conducted under dim red lighting to minimize any uncontrolled photorepair activity.

### 2.6. CPD Analysis

To determine the steady-state levels of CPD in rice plants, DNA extraction, preparation of agarose plugs, and treatment of DNA samples with a UV endonuclease was performed as previously detailed.^16^

The CPD frequencies were determined using a DNA damage analysis system constructed by Tohoku Electric Co. (Miyagi, Japan), as described previously.^32^ The CPD frequencies were calculated using a molecular length standard curve and the quantity of DNA at each migration position, as indicated by the quantitative image data.^33^ CPD frequencies are expressed in units of CPD Mb^−1^.

### 2.7. Statistical Analysis

All experiments were repeated at least in triplicate. The data were analyzed using Microsoft Office Excel 2016 (Microsoft Co., Redmond, WA, USA) and GraphPad Prism v8.00 (GraphPad Software, San Diego, CA, USA). Statistical significance of differences was calculated using the Tukey-Kramer test with a confidence level of 95.0% (*, a, b, c, d, e, f, *P* < .05).

## 3. Results

### 3.1. Generation of CPD Photolyase-transgenic African Rice (O. Glaberrima)

**Figure 1.**
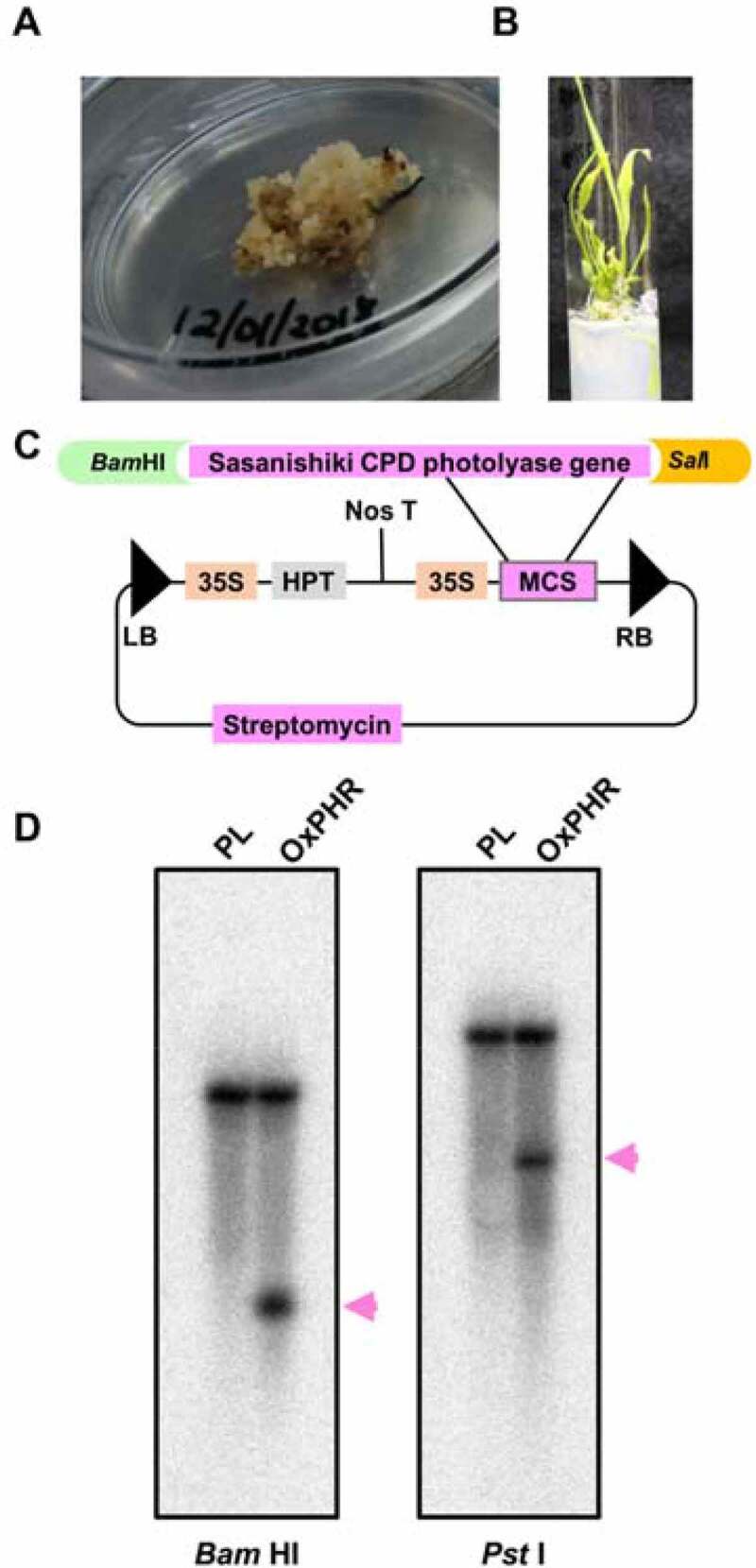
Generation of the cyclobutane pyrimidime dimer (CPD) photolyase-overexpressing transgenic African rice plant (TOG12380-OxPHR). (a) Hygromycin-selected callus of TOG12380. (b) Successfully regenerated callus of the cultivar TOG12380 on Murashige and Skoog (MS) media. (c) The transformation construct pPZP2Ha3 that was used to transform rice with cDNA encoding the CPD photolyase of the Sasanishiki cultivar. This cDNA was subcloned into a multi-cloning site (MCS) of the binary vector pPZP2Ha3 in the sense orientation. (d) Southern blot analysis of the rice CPD photolyase gene in the parental line (PL) and overexpressing transgenic plants (OxPHR). Genomic DNA (20 μg) isolated from PL and OxPHR plants was digested with *Bam*HI or *Pst*I and then separated on 0.8% agarose gel. Following its transfer to a nylon membrane, the DNA blot was hybridized with a ^32^P-labeled CPD photolyase gene probe. The magenta arrowheads point to the band of the CPD photolyase transgene.

In general, to achieve the genetic transformation and generation of transgenic rice plants, a routine tissue culture system that includes callus induction and plantlet regeneration is a fundamental requirement. There have been reports on the successful production of transgenic African rice.^19,34^ However, because an appropriate routine tissue culture protocol for African rice cultivars, including *O. glaberrima*, is yet to be established, the production of transgenic African rice remains a challenge.^20–23^ Thus, we first attempted to induce calli from four African rice cultivars (Jiakawo Wodewo, TOG14928, TOG12380 [*O. glaberrima*], and TOB7307 [*O. barthii*]) examined in our previous UVB sensitivity study,^10^ along with Nipponbare (*O. sativa*) as a control, by using several media for callus induction (Table S1). When using a medium found suitable for inducing the callus in Nipponbare, calli were not induced by TOG14928 or TOG12380, but small brown calli were slightly induced from both Jiakawo Wodewo and TOB7307 (Table S1). We changed the concentration and type of callus-inducing hormone, but no callus was induced in African rice cultivars when using an N6-based medium (Table S1). Therefore, we shifted to an MS-based medium and, consequently, white calli with a high growth rate were induced from TOG12380 (*O. glaberrima*) (Fig. 1a). Using the same medium, calli were induced not only from TOG12380 but also from the three cultivars of *O. glaberrima* (TOG14928, MB3, and C7251) and *O. barthii* (TOB7307), but not from one cultivar of *O. glaberrima* (Jiakawo Wodewo) (Fig. 1a, Table S1). In summary, the medium deemed suitable for calli induction varied among African rice cultivars and was strongly dependent on their genotype. However, we succeeded in inducing calli from four of five *O. glaberrima* and one *O. barthii* by using MS medium containing 10 µM 2,4-D.

Next, we investigated the suitable medium conditions for regeneration by changing the relative concentrations of sucrose and hormones based on the MS medium, as shown in Table S2. We found that modifying the MS media by increasing the sucrose concentration to 50 g L^−1^ and changing the optimal hormonal concentration to 0.1 µM 2,4-D with 50 µM kinetin (Table S2) resulted in successful regeneration via calli of the *O. glaberrima* cultivar TOG12380 (Fig. 1b). The calli induced from four *O. glaberrima* were used for regeneration testing; however, this failed for MB5, C7251, and TOG14928 (Table S2). Therefore, only the successfully regenerated TOG12380 was used for subsequent experiments.

Next, we attempted to generate transgenic rice plants that overexpressed the CPD photolyase using TOG12380 (*O. glaberrima*), which shows UVB sensitivity, similar to the wild type. The sense cDNA of the CPD photolyase of Sasanishiki (*O. sativa*), a *japonica* rice cultivar with higher CPD photolyase activity, was subcloned into the binary vector pPZP2Ha3 with a hygromycin phosphotransferase gene (*hpt*) as a selection marker under the control of the cauliflower mosaic virus 35S promoter (Fig. 1c), as described previously.^17^ The subclone was transferred into *Agrobacterium* and infected into the calli of the UVB-sensitive TOG12380 strain. As a result, we successfully isolated one independent hygromycin-resistant transgenic line (T_0_ plant) overexpressing the Sasanishiki CPD photolyase (TOG12380-OxPHR line) from the parental line TOG12380 (TOG12380-PL) (Fig. S2). The transformation efficiency of TOG12380 was 1.4%. The self-fertilized plants (T_1_) of T_0_ plants were obtained and sown in a hygromycin-containing medium. If the transgene was a single copy, the segregation ratio of the transgene was expected to match the expected Mendelian ratio of 3:1 (three hygromycin-resistant plants and one susceptible plant). In T_1_ plants, 23 of the 35 plants showed hygromycin resistance, and the resistance percentage was 66%. Self-fertilized plants (T_2_) were generated from 12 hygromycin-resistant T_1_ plants. Three out of 12 progenies showed 100% hygromycin resistance, while the others showed 68–88% hygromycin resistance. One of the three progenies which showed 100% hygromycin resistance in T_2_ seeds or T_3_ produced by self-fertilization of T_2_ was used as a homozygous TOG12380-OxPHR line for further analyses. To determine the copy number of the transferred CPD photolyase gene, the TOG12380-OxPHR line was analyzed by southern blot analysis,^35^ in which genomic DNAs isolated from T_2_ were digested by restriction enzymes *Bam*HI and *Pst*I (Fig. 1d). The copy number of the CPD photolyase gene in the TOG12380-OxPHR line was one (Fig. 1d).

### 3.2. Characteristics of Transgenic Rice: Transcript Levels and in Vitro and in Vivo CPD Photolyase Activity

**Figure 2.**
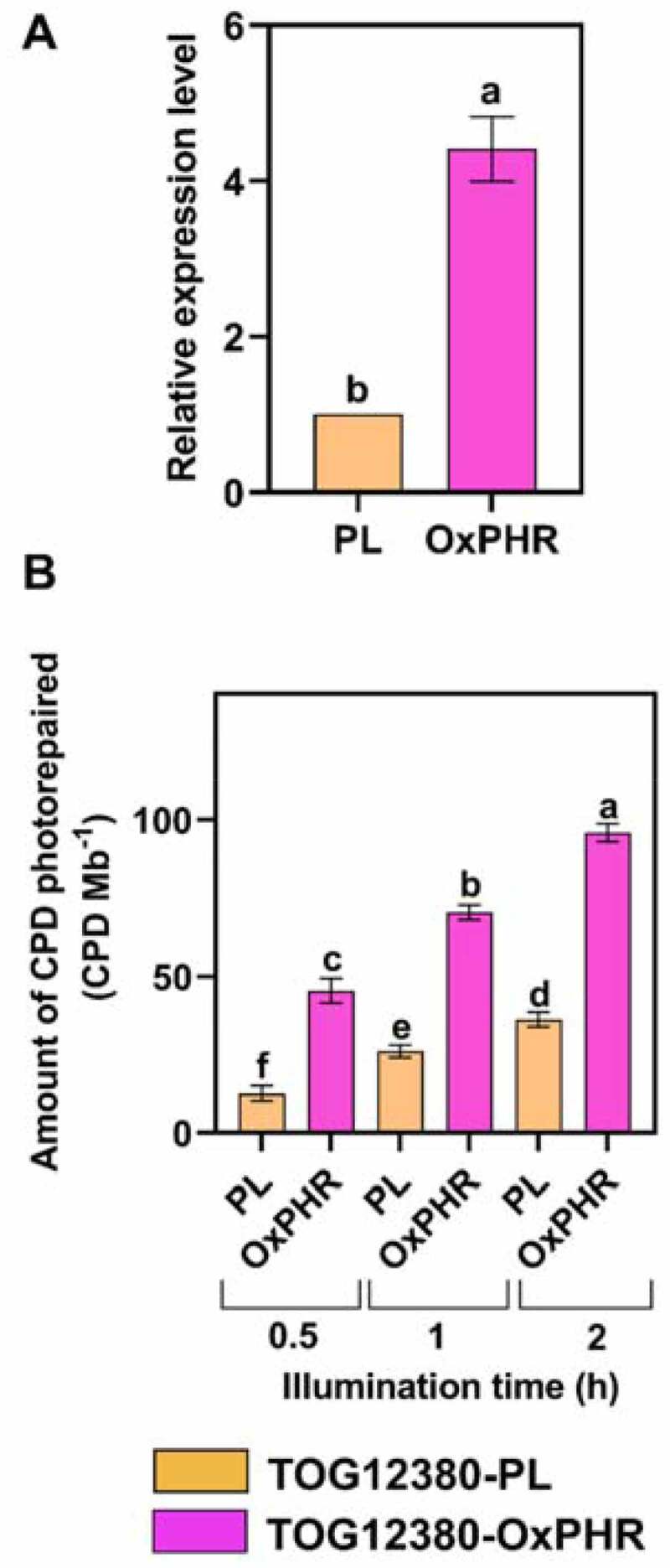
The CPD photolyase transcript levels and *in vitro* CPD photolyase activity of the overexpressing transgenic (TOG12380-OxPHR: pink bar) and parental (TOG12380-PL: orange bar) rice plants. (a) Ratios of CPD photolyase transcripts (measured by quantitative real-time RT-PCR analysis) of OxPHR transgenic plants relative to those of the PL plants. Actin served as an internal control. The level of PL was set to 1. (b) Amount CPD photorepaired by the crude soluble protein of TOG12380-OxPHR and TOG12380-PL. Crude extracts of the fully expanded 3rd leaves of each experimental plant were mixed with UV-irradiated λDNA, then incubated in the dark for 15 min at 30°C to facilitate a photolyase-CPD complex formation, after which they were exposed to continuous blue light for 0.5, 1, and 2 h. Values are mean ± SD, for n = 4 replicates in (A) and (B); different letters indicate significant differences determined by the Tukey-Kramer test (*P < *.05).

We first investigated the extent to which the CPD photolyase transcript level and activity increased in the TOG12380-OxPHR plant. For this, TOG12380-OxPHR and PL plants were grown under visible radiation alone. Then, the 3rd leaves of their 13-day-old seedlings were analyzed. First, the CPD photolyase transcripts in TOG12380-OxPHR and PL plants were determined using quantitative real-time RT-PCR. The CPD photolyase transcript level, calculated as the ratio of CPD photolyase transcripts of the TOG12380-OxPHR plant to that of the PL plant, was 4.4-fold higher in the TOG12380-OxPHR plant than in the TOG12380-PL (Fig. 2a). Next, the *in vitro* CPD photolyase activity in the crude extracts of the 3rd leaves of the TOG12380-OxPHR line and PL was measured using UV-irradiated λDNA as a substrate. The CPD frequencies were calculated by an assay with alkaline agarose gel electrophoresis.^16^ In the reaction mixture with the TOG12380-OxPHR crude extract, the amount of CPD photorepaired was higher within 2 h under blue light exposure than in the mixture containing the PL crude extract (Fig. 2b). Thus, the *in vitro* CPD photorepair activity was significantly higher in the leaf cell extract from TOG12380-OxPHR than PL; approximately 2.6-fold higher in TOG12380-OxPHR than in PL plants (Fig. 2b).

**Figure 3.**
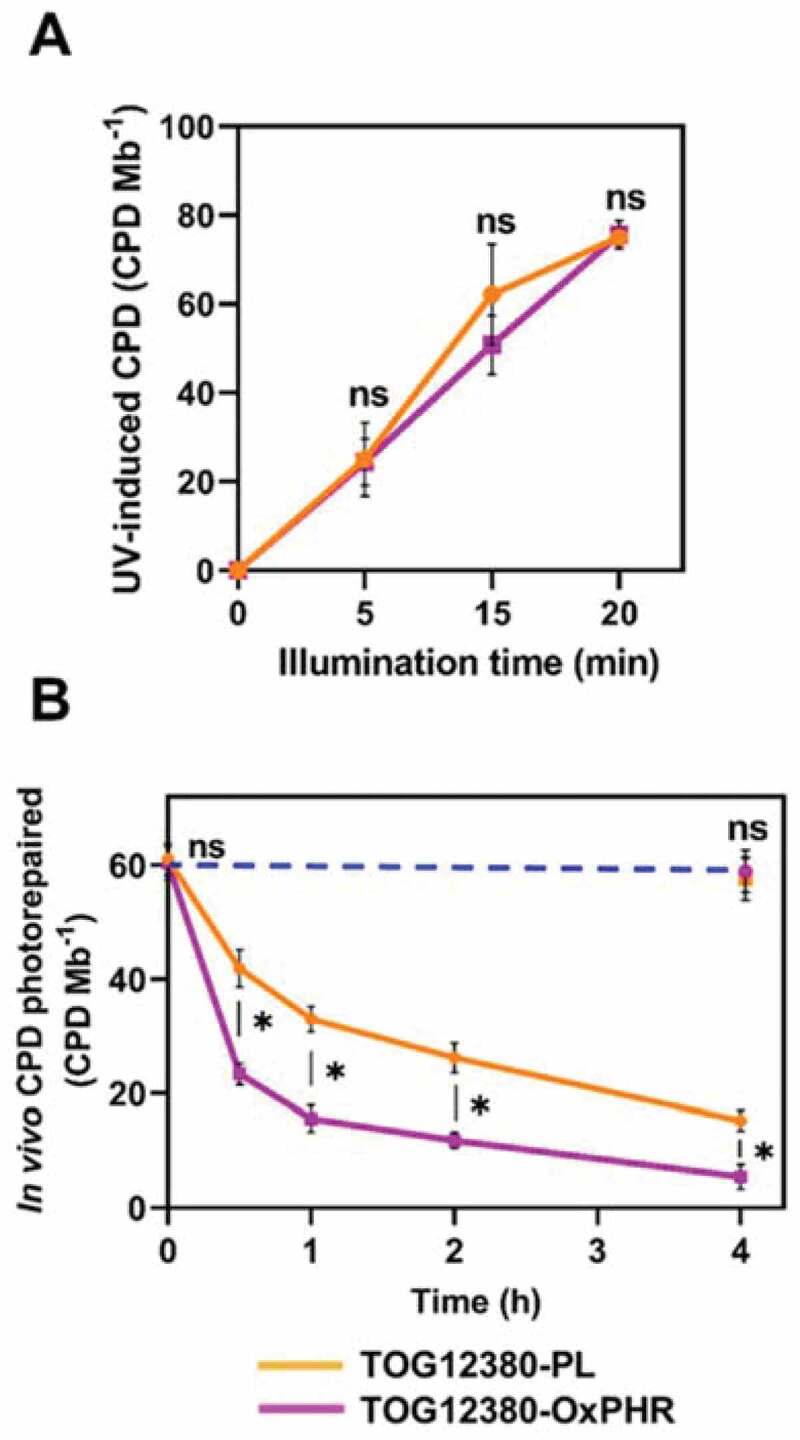
Susceptibility to UV-induced CPD and *in vivo* CPD photorepair activities of the TOG12380-OxPHR (pink line) and TOG12380-PL (orange line) rice seedlings. (a) Susceptibility to UV-induced CPD. The detached 3rd leaves were exposed to UV radiation (germicidal lamp) at a distance of 10 cm for 0–20 min, and their DNA was isolated and their CPD levels determined. (b) *In vivo* CPD photorepair activities. The detached 3rd leaves were exposed to UV radiation for 15 min, to induce approximately 60 CPD Mb^−1^, and then exposed to blue light (60 μmol m^−2^ s^−1^) for various durations (0, 0.5, 1, 2, and 4 h) or kept in the dark for 4 h (dotted blue line). Samples were harvested at the indicated times, and then the CPD frequencies in their respective DNA was measured. Values are mean ± SD for n = 3 replicates in (A) and (B); asterisks indicate a significant difference determined by the Tukey-Kramer test, ns = not significant (*P < *.05).

Although the *in vitro* CPD photolyase activity in the cell extract of TOG12380-OxPHR was enhanced compared to that of the PL plant, the actual UVB resistance of rice plants depends on their susceptibility to CPD induction via UVB exposure.^16,17^ Therefore, to examine these characteristics, the susceptibility of the transgenic and PL plants to UV-induced CPDs was determined by exposing the detached 3rd leaves to UV radiation for different time periods to induce CPDs. Their DNA was isolated and the corresponding CPD levels were measured. The CPD levels increased with a longer duration of UV illumination, with no significant difference detected in the dose-response curves between the TOG12380-OxPHR and PL plants (Fig. 3a). Thus, both TOG12380-PL and TOG12380-OxPHR have similar susceptibilities to CPD induction. Next, the *in vivo* ability of the plants to repair CPDs under blue light was examined by exposing the 3rd leaves of the plants to UV radiation for 15 min to induce an estimated 60 CPD Mb^−1^, followed by illumination with blue light for different lengths of time. The degree of CPD photorepair revealed a significant improvement in the photorepair activity of TOG12380-OxPHR seedlings compared with that of PL (Fig. 3b). The OxPHR seedlings repaired approximately 37.2 CPD Mb^−1^ in 0.5 h, while the PL seedlings repaired only about 17.4 CPD Mb^−1^. These results suggest that the OxPHR transgenic plants had increased cellular CPD photolyase activity compared to PL plants.

### 3.3. UVB Resistance of Transgenic Rice and Steady-state CPD Analysis

To test the resistance of transgenic TOG12380-OxPHR to UVB radiation of various intensities, the growth of TOG12380-PL and TOG12380-OxPHR were examined for 21 days in a growth chamber under visible radiation alone or with supplementary various UVB_BE_ of 4.9, 9.8, or 14.7 kJ m^−2^ per day, which was adjusted just above the plants. In this experiment, we used the high UVB intensity of 1.2 W/m^2^ (14.7 kJ m^−2^ day^−1^), similar to our previous study,^10^ to clearly distinguish phenotypical differences between UVB resistance and sensitive plants. On the other hand, the lower UVB intensity of 0.8 W m^−2^ (9.8 kJ m^−2^ day^−1^) and 0.4 W/m^2^ (4.9 kJ m^−2^ day^−1^) were used to the mimic outside ground level of UVB radiation. The average of UVB_BE_ at ground level in a year at Sonoran Desert (USA) or Cape town (South Africa) is 8.0 kJ m^−2^ d^−136^ or 8.5 kJ m^−2^ d^−1^,^37^ respectively.

**Figure 4.**
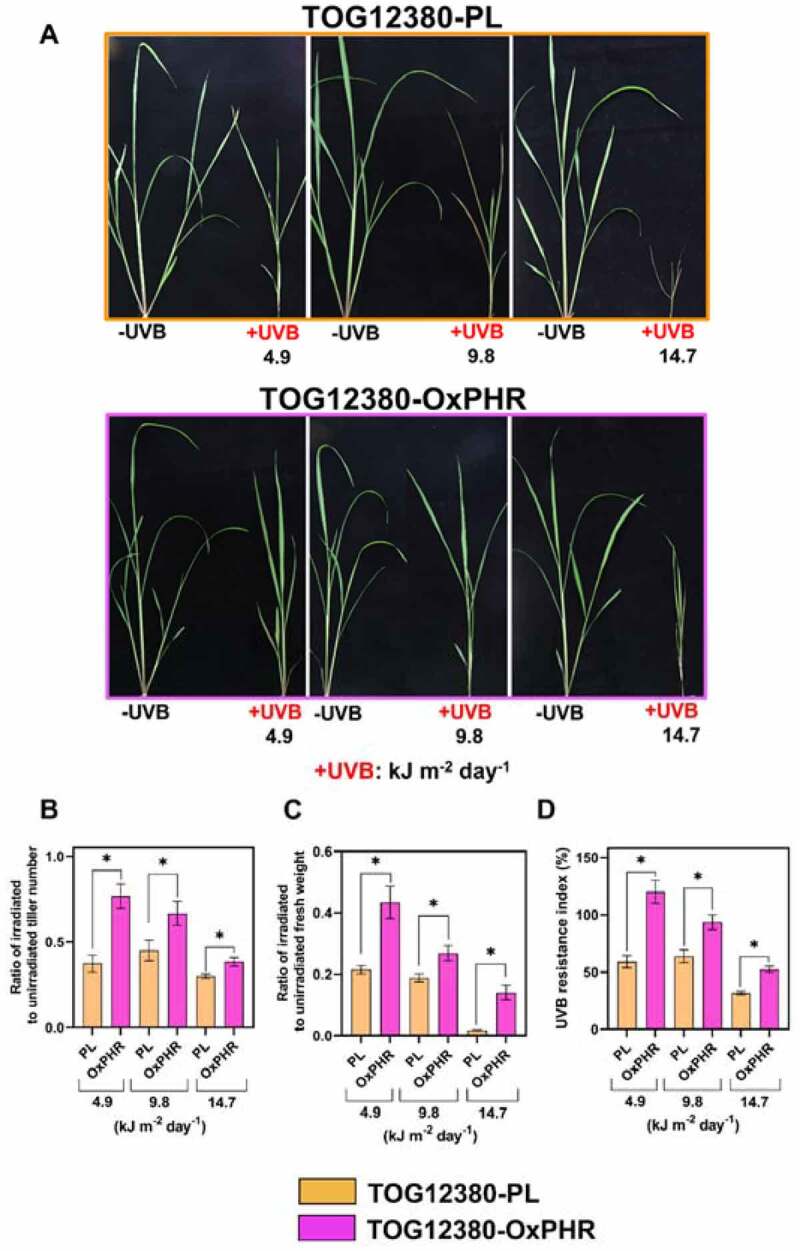
Effects of supplementary UVB radiation on the growth of TOG12380-OxPHR and TOG12380-PL rice plants. (a) Photographs of plants grown for 21 days under visible radiation supplemented with [(+UVB); 4.9, 9.8, and 14.7 kJ m^−2^ day^−1^] or without (−UVB) UVB radiation. (b) Ratio of irradiated to unirradiated tiller number (+UVB)/(−UVB). (c) Ratio of irradiated to unirradiated aboveground fresh weight of plants. (d) The UVB resistance index was determined by summing the value of the ratio of +UVB to −UVB of tiller number and aboveground fresh weight, and multiplying it by 100, i.e., (+UVB)/(−UVB) × 100. Values are mean ± SD based on three independent experimental replicates (with n = 9 or 10), each performed in triplicate in (b–d); orange bar; TOG12380-PL, pink bar; TOG12380-OxPHR, the asterisk indicates significant differences determined by the Tukey-Kramer test (*P < *.05).

For rice plants, the tiller number and fresh weight (FW) of their aboveground parts are mostly correlated with grain yield.^38,39^ Therefore, the effect of supplemental UVB radiation on the tiller number and FW of the aboveground parts of the OxPHR and PL lines were investigated. Under visible radiation alone, there was no significant difference among the plant types in their tiller numbers, although the FW of TOG12380-OxPHR was lower than that of TOG12380-PL (Fig. 4a, Table S3). In contrast, the transgenic TOG12380-OxPHR plants grew significantly better than the TOG12380-PL plants when exposed to various intensities of supplementary UVB radiation. The UVB resistance index was determined by multiplying the sum of two ratios by 100: (1) irradiated to unirradiated tiller number and (2) irradiated to unirradiated FW.^40^ The UVB resistance index was significantly higher in TOG12380-OxPHR compared to TOG12380-PL; the index of TOG12380-OxPHR at 4.9, 9.8, and 14.7 kJ m^−2^ d^−1^ UVB_BE_ were 120, 90, and 52, respectively, while the index of TOG12380-PL at 4.9, 9.8, and 14.7 kJ m^−2^ d^−1^ UVB_BE_ were 57, 61, and 32, respectively (Fig. 4d, Table S3). These findings indicate that, compared with PL plants, the UVB-sensitive African rice (*O. glaberrima*) overexpressing CPD photolyase was much more resistant to the growth-inhibiting effects of UVB radiation.

**Figure 5.**
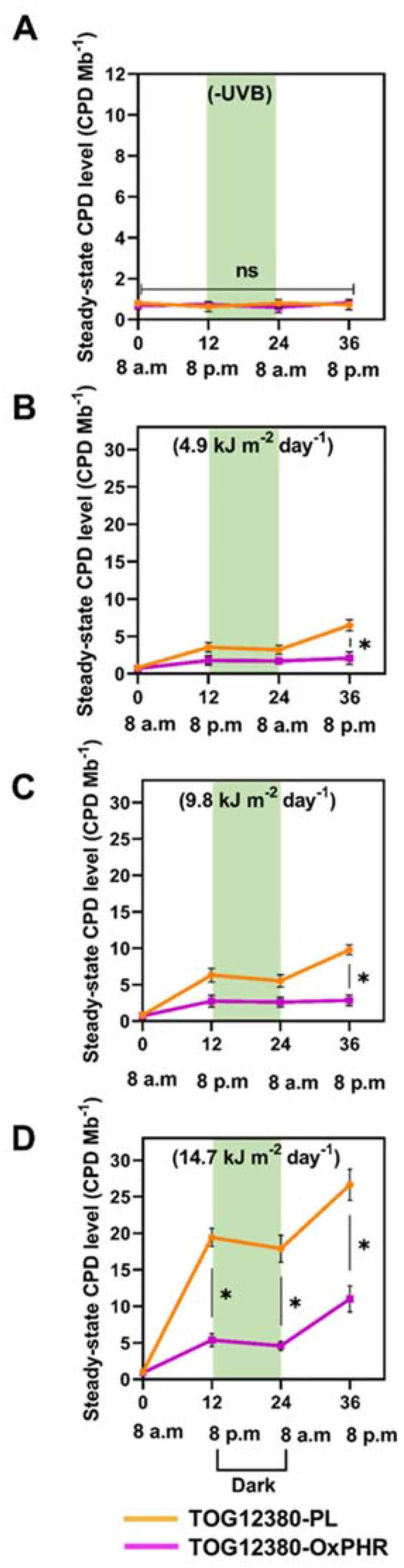
Changes in steady-state CPD levels in the 3rd leaves of TOG12380-OxPHR (pink line) and TOG12380-PL (orange line) rice plants grown with or without supplementary UVB radiation. (a) Changes to the steady-state CPD levels in transgenic and PL plants growing without supplementary UVB radiation. (b–d) Changes to the steady-state CPD levels in transgenic and PL plants grown with supplementary UVB radiation. Plants were first grown under visible radiation without supplementary UVB radiation in a large growth cabinet until their 3rd leaves had expanded fully. Then, some of the potted plants were grown under visible radiation with (b) 4.9 kJ m^−2^ day^−1^, (c) 9.8 kJ m^−2^ day^−1^, and (d) 14.7 kJ m^−2^ day^−1^ supplementary UVB radiation. Over the next 2 days, from groups of plants their 3rd leaves were detached after every 12 h (8 AM to 8 PM on the first day, 8 PM to 8 AM, and 8 AM to 8 PM of second day). The CPD levels in the leaf DNAs were then determined. Values are means ± SD for n = 3 replicates; asterisks indicate significant differences determined by the Tukey-Kramer test (*P < *.05), ns = not significant. The pale-green shaded area indicates the dark treatment.

Moreover, we empirically confirmed steady-state CPD levels in the leaves of transgenic and PL plants grown with or without supplementary UVB radiation. These plants were grown under visible radiation until the 3rd leaf had expanded fully, after which some of the potted plants were grown under visible radiation supplemented with UVB radiation at 4.9, 9.8 or 14.7 kJ m^−2^ d^−1^ UVB_BE_. When grown under visible radiation only, the steady-state CPD levels were similar between the TOG12380-OxPHR and PL plants, remaining below 1.0 CPD Mb^−1^ during the day and night (Fig. 5a). Yet when the PL plants grew under supplementary UVB radiation at 4.9 kJ m^−2^ d^−1^ UVB_BE_, their CPD levels increased from 1 to 3.5 CPD Mb^−1^ on the first day (0–12 h); these levels remained the same during the night and the following day, but started to rise again at 8 AM on the second day (onset of illumination), reaching 6.5 CPD Mb^−1^ (Fig. 5b). A similar trend was observed in the leaves of the PL plants grown under supplementary UVB radiation at 9.8 or 14.7 kJ m^−2^ d^−1^ UVB_BE_, whose CPD levels increased depending on the UVB_BE_ intensity they were exposed to (Fig. 5c, d). By contrast, the steady-state CPD level of the TOG12380-OxPHR plants, growing under UVB_BE_ intensity of 4.9 kJ m^−2^ d^−1^, was significantly lower than that of TOG12380-PL, remaining below 2.0 CPD Mb^−1^ during the day and night (Fig. 5b). Similarly, for TOG12380-OxPHR plants grown under supplementary UVB radiation at 9.8 or 14.7 kJ m^−2^ d^−1^ UVB_BE_, their steady-state CPD levels were significantly lower than those of TOG12380-PL counterparts (Fig. 5c, d). These results indicate that a low CPD level in TOG12380-OxPHR grown under various supplementary UVB radiation conditions was caused by high CPD photolyase activity. These results strongly suggest that, despite *O. glaberrima* being a species different from *O. sativa*, UVB-induced CPDs are still one of the main causes of UVB-induced growth impairment in rice plants grown under supplementary UVB radiation. Thus, enhancing CPD photolyase activity can confer greater resistance to UVB radiation, probably by reducing the amount of CPD in the cells.

## 4. Discussion

Although transforming and regenerating several African rice varieties (*O. glaberrima* and *O. barthii*) was challenging (Table S1 and S2), we succeeded in introducing the CPD photolyase gene into one strain among the *O. glaberrima* investigated here (Fig. 1, S2). The difficulties in transforming *O. glaberrima* cultivars have been previously documented by others^20–22^; however, information on the callus induction and plantlet regeneration of *O. glaberrima* plants is limited.^41^ In this study, we first introduced the CPD photolyase gene using an immature embryo instead of a mature seed, since the former is often deemed an effective method for introducing a targeted gene without callus induction.^42,43^ The protocol using an immature embryo worked well only for the *japonica* rice cultivar Nipponbare (*O. sativa*) (Fig. S1B), but not for the African rice cultivar TOB7307 (Fig. S1A). Therefore, we induced calli from African rice cultivars and fortunately derived a medium deemed suitable for callus induction from African rice varieties (Table S1). Furthermore, in a modification of the media following Brisibe et al. (1990), increasing its concentration of sucrose to 50 g L^−1^ enabled the successful regeneration of the UVB-sensitive African cultivar TOG12380 (Fig. 1b).^23^ Although calli induced from five African rice cultivars were used in the regeneration testing, only the callus from TOG12380 was regenerated (Table S2, Fig. S1C, D). Failed shoot regeneration was also observed in the callus of the cultivar IRGC104165 (*O. glaberrima*).^22^ Therefore, although further studies are needed to improve the regeneration of African rice cultivars, the information provided here on the media conditions for callus induction and regeneration, which are important for the generation of transgenic rice, will be of great help in the development of protocols for future transgenic African rice production.

Our prior studies demonstrated that enhancing CPD photolyase activity increases UVB resistance in *O. sativa* species.^17,18^ However, it was unclear whether the overexpression of this gene in African rice (*O. glaberrima*), a different species in the rice plant genus, would also increase UVB resistance. To the best of our knowledge, no study has yet developed a UVB-resistant transgenic line of *O. glaberrima*, although most African rice cultivars are known to be highly sensitive to UVB radiation.^10^ The generated UVB-resistant transgenic African rice cultivar (TOG12380-OxPHR) was capable of increasing CPD photolyase activity, achieved through ubiquitous expression of the CPD photolyase gene from Sasanishiki, with higher CPD photolyase activity facilitated by the CaMV 35S promoter (Fig. 1c). The TOG12380-OxPHR plant type carried a single copy of the CPD photolyase enzyme for DNA repair (Fig. 1d), with a 4.4-fold higher level of CPD photolyase transcripts and 2.6-fold higher activity level than its PL counterpart (Fig. 2). Similar to the PL and OxPHR generated previously in Asian rice,^17^ both PL plants and the TOG12380-OxPHR generated in this study showed equivalent susceptibility to UV-induced CPD formation, but the TOG12380-OxPHR CPD levels were significantly reduced when grown under supplementary UVB radiation (Fig. 5). These results strongly suggest that, despite the species barrier, UVB-induced CPDs are the main cause of UVB-induced growth inhibition in rice plants grown under supplementary levels of UVB radiation stress. Accordingly, enhancing CPD photolyase activity may significantly alleviate UVB-induced growth inhibition in rice crops.

Rice varieties cultivated in tropical areas, where the amount of UVB is relatively high, typically display UVB sensitivity compared with rice varieties cultivated in temperate areas; Surjamkhi^6^ and Kasalath^9^ have been domesticated in the tropical areas of Bengal. Most African rice cultivars (of *O. glaberrima* and *O. barthii*) are also highly sensitive to UVB radiation (i.e., are UVB-super-hypersensitive) than the rice cultivar Surjamkhi.^10^ The fact that cultivars with a very high sensitivity to UVB radiation have been domesticated and are still cultivated today in tropical areas of Bengal and Africa suggests that their UVB-sensitive traits may be beneficial to them. UVB radiation can improve or reduce the resistance of plants to other environmental stresses, such as pathogens.^44,45^ Although there may be crosstalk between UVB sensitivity and other plant responses to environmental stresses, whether this depends on UVB-induced CPD remains unclear. Because the TOG12380-OxPHR and PL plants have the same genetic background but differ in their CPD photolyase activities, we encourage future studies to use these two plant types to elucidate whether UVB-induced CPD affects rice tolerance to other environmental stresses.

Finally, due to the fact that the effects of climate change and growing populations are most pronounced in Africa, which receives more UVB radiation via sunlight than other continents, there is increased pressure to identify short-term solutions to raise the crop yields and agriculture sustainability here. Thus, breeding African rice with the capacity for high CPD photorepair is a promising approach to strengthen our knowledge toward developing UVB-tolerant African rice cultivars that can maintain high productivity. However, although we have generated UVB-resistant transgenic African rice, this study only examined the effect of elevated UVB radiation on the growth of UVB-resistant transgenic African rice, *O. glaberrima*, grown in a growth chamber. Therefore, future studies on the effect of increasing CPD photolyase activity on the growth and yield of rice grown outdoors under natural sunlight should be conducted. Since African rice is grown in a tropical climate with high levels of UVB radiation from sunlight, this study provides important targets for improving the resistance to UVB by manipulating the CPD photolyase gene, which, if capitalized upon, could contribute to food security by helping to feed a growing world population, especially in Africa.

## 5. Conclusion

The establishment of genetic engineering for plants is important not only for the creation of plants resistant to various stresses, but also for the elucidation of gene functions. In this study, we succeeded in generating a UVB-resistant transgenic African rice plant with high CPD photolyase activity. These results prove not only the bioengineering of UVB-resistant African rice, but also the important role of CPD photolyase in generating UVB-resistant African rice crops. Therefore, CPD photolyase is an excellent candidate for the alleviation of UVB-induced damage through conventional breeding or bioengineering in African rice. Today, plants are being damaged by various environmental stresses due to drastic changes in climate around the world, including Africa. To solve this problem, it is necessary to establish genetic recombination technology for various plant crops, including African rice, in the future. The results and information obtained in this study will greatly contribute to the development of protocols for producing transgenic African rice.

## Supplementary Material

Supplemental Material
