# Supplementary material for: Transgenic rice Oryza glaberrima with higher CPD photolyase activity alleviates UVB-caused growth inhibition
Source: GM Crops Food. 2021 Dec 22;12(1):435–48. doi: 10.1080/21645698.2021.1977068 (PMC8820246; doi:10.1080/21645698.2021.1977068)
Supplement: Supplemental Material [file KGMC_A_1977068_SM8364.docx]

**<Supplementary Materials and Methods>**

**Plant materials**

To improve callus induction and regeneration in African rice (*O. glaberrima*), *O. sativa* ssp. *japonica* cv. Nipponbare was used as a positive control. Several African rice varieties, namely TOG12380, TOG14928, MB3, C7251, Jiakawo Wodewo (*O. glaberrima*), and TOB7307 (*O. barthii*), were used in this experiment. All of them, except C7251, were provided by the Rice Biodiversity Center for Africa (http://eservices.africarice.org/argis/index.php; Cotonou, Benin). C7251 was obtained from the Genetic Strain Research Center, National Institute of Genetics (Mishima, Japan).

**Seed preparation and sterilization**

Mature rice seeds were dehusked manually, followed by sterilisation using 70% ethanol for 1 min. Next, the ethanol was discarded, and the seeds were rinsed twice with sterilised water. Seeds were then placed on a mixture of Clorox (effective chlorine concentration of 2%) with Tween-20 (2 or 3 drops for a 50-ml solution), and shaken on an orbital shaker at 120 rpm for 20 min. The sterilised seeds were then rinsed four times with sterile water. These surface-sterilized seeds were then ready for callus induction.

**Induction of embryogenic callus**

Four African rice cultivars, TOG12380, TOG14928, Jiakawo Wodewo (*O. glaberrima*), TOB7307 (*O. barthii*), and Nipponbare (*O. sativa*), were used to induce embryonic callus formation. Although we screened the best callus induction media for all rice varieties, including N6 ^46^, N6-Surjamkhi ^18^, and MS ^23^, under various hormonal combinations, including 9.1, 10, and 13.6 µM of 2,4-D and 30 µM of dicamba ^23^ (composition of these media can found below), resulted to very good callus on cultivar TOG12380 (*O. glaberrima*) (Table S1). Subsequent experiments focused on TOG12380 using MS-CI as the callus induction medium. Surface-sterilized seeds were sown on MS-CI media. Then, petri dishes containing 5 or 6 seeds were sealed with surgical tape, followed by incubation under fluorescent light at 30°C. Depending on the freshness of the seeds, calli were produced for approximately 30 days.

**Agrobacterium infection and co-cultivation**

The procedure for constructing the vector is described in detail in the Materials and Methods. Briefly, *Agrobacterium tumefaciens* strain EHA101 ^26^ and binary vector pPZP2Ha3 ^24^ were used for plant transformation. *Agrobacterium* (strain EHA101), which retained the transform construct pPZP2Ha3, were grown at 28°C, for three days, on yeast extract peptone (YEP) medium plates supplemented with 0.05 mg L^-1^ of hygromycin and 0.05 mg L^-1^ of kanamycin (EHA101 harbours kanamycin resistance). *Agrobacterium* (EHA101) cells were pelleted and resuspended in 50 ml of AAM liquid infection medium. Finally, using the same AAM liquid infection medium, the *Agrobacterium* suspension cell was diluted to OD_600_ 0.05–0.1. To enhance the transformation, prior to the infection process, the calli were subcultured on fresh MS-CI medium for 3 to 4 days ^47^. Afterwards, subcultured calli were soaked in bacterial suspensions and gently shaken for 1 min. The excess bacteria were removed by pouring out the infection medium and by blotting the calli with sterilised Kimwipes. For co-cultivation, infected calli were placed on a N6-AS co-cultivation medium ^47^ containing sterile filter paper discs placed between the calli and the surface of the medium to prevent overgrowth of *Agrobacterium*. The calli and bacterial cells were co-cultivated at 28°C for 3 days in the dark.

**Selection of transformed calli**

On a clean bench, the infected calli were moved into a sterile container and then washed by shaking at least seven times with sterile water until water appeared clear. During the final wash, carbenicillin (400 mg L^-1^) was added to the washing solution. Later, the calli were dried briefly on sterilised Kimwipes paper. These calli were then transferred to an MS-S selection medium containing 50 mg L^-1^ of hygromycin and 400 mg L^-1^ of carbenicillin, and incubated at 30°C under fluorescent light for about 3 to 4 weeks. Approximately 30 calli were placed on each plate dish (90 mm diameter) and observed for almost 1 month until hygromycin-resistant calli grew on the plate.

**Plant regeneration and acclimatization**

Before starting the regeneration of transformed calli, we screened for the best callus regeneration medium using non-transformed calli. Calli were induced from five African rice cultivars, namely TOG12380, TOG14928, MB3, C7251 (*O. glaberrima*), and TOB7307 (*O. barthii*), using MS-CI medium. As noted by Brisibe et al. (1990), 5% sucrose resulted in a high frequency of shoot formations ^23^. We adapted this condition by variously modifying the hormone concentration (auxin and cytokinin) and increasing the sucrose concentration to 7% ^41^, with the aim of enhancing the regeneration efficiency (Table S2). TOG12380 was successfully regenerated from calli via the regeneration medium containing 5% sucrose, 0.1 µM of 2,4-D and 50 µM of kinetin (MS-RE regeneration medium). Therefore, the selected hygromycin-resistant calli were transferred to an MS-RE regeneration medium containing 50 mg L^-1^ hygromycin and 400 mg L^-1^ carbenicillin. The calli were then kept at 30°C under continuous fluorescence light for approximately 1 month. After a green shoot(s) of the cultivar TOG12380 was observed, a regenerated shoot was transferred to MS-RI root induction media containing 50 mg L^-1^ hygromycin. After 2 weeks, the ensuing plantlet was gently washed with water to remove the adhering medium, thereby minimising possible fungal attacks. Subsequently, a plantlet with its healthy roots was transplanted into soil in a pot, and the transplanted plant was covered with an open-top plastic tube (40 cm in height) for the acclimatisation phase. Plants were grown under visible radiation in a growth chamber (ESPEC MIC CORP., Osaka, Japan) with a 12-h/12-h photoperiod and corresponding temperatures of 27°C/17°C. After the growth was confirmed, the tube cover was removed.

**Media**

All the media solutions were made based on N6 ^46,47^ and MS ^23,27^.

**Stock solutions**

MS stock 1 (vitamins) stock (1000× stock)

1 g L^-1^ of thiamine HCl and 200 g L^-1^ of *myo*-inositol

Mn, Zn, KI, H_3_BO_3_ stock (1000× stock)

10 g L^-1^ of MnSO_4_**^.^**4-5H_2_O, 2.0 g L^-1^ of ZnSO_4_**^.^**7H_2_O, 0.75 g L^-1^ of KI, 3.0 g L^-1^ of H_3_BO_3_

Cu, Co, Mo stock (1000× stock)

25 mg L^-1^ of CuSO_4_**^.^**5H_2_O, 25 mg L^-1^ of C_O_Cl_2_**^.^**6H_2_O, 250 mg L^-1^ of Na_2_MoO_4_**^.^**2H_2_O

AAM vitamins stock (1000× stock)

7.5 g L^-1^ of glycine, 100 g L^-1^ of *myo*-inositol, 1.0 g L^-1^ of nicotinic acid, 1.0 g L^-1^ of pyridoxine HCL, 10 g L^-1^ of thiamine HCl

N6 stock 1 (100×)

46.3 g L^-1^ of (NH_4_)_2_SO_4_, 185 g L^-1^ of MgSO_4_**^.^**7H20, 40 g L^-1^ of KH_2_PO_4_

N6 stock 2 (100×)

16.6 g L^-1^ of CaCl_2_**^.^**2H_2_O

N6 stock 3 (1000×)

4.4 g L^-1^ of MnSO_4_**^.^**4-5H_2_O, 1.5 g L^-1^ of ZnSO_4_**^.^**7H_2_O, 0.8 g L^-1^ of KI, 1.6 g L^-1^ of H_3_BO_3_

N6 stock 4 (1000×)

37.3 g L^-1^ of Na_2_EDTA

N6 stock 5 (1000×)

27.8 g L^-1^ of FeSO_4_**^.^**7H_2_O

N6 stock 6 (1000×)

2 g L^-1^ of glycine, 100 g L^-1^ of *myo*-inositol, 0.5 g L^-1^ of nicotinic acid, 0.5 g L^-1^ of pyridoxine HCl, 1 g L^-1^ of thiamine HCl

**MS callus induction and subculture (MS-CI) medium**

MS-salt ^27^ (FUJIFILM Wako Pure Chemical Corporation, Osaka, Japan), MS stock 1 (1×), with 10 µM 2,4-D, 78.4 mg L^-1^ Fe-EDTA, 3 g L^-1^ casamino acid, 30.0 g L^-1^ sucrose, and 0.8% agar (pH 5.8).

**AAM liquid infection medium**

250 mg L^-1^ of MgSO_4_.7H_2_O, 150 mg L^-1^ of CaCl_2_.2H_2_O, 150 mg L^-1^ of NaH_2_PO_4_.2H_2_O, 40 mg L^-1^ of Fe-EDTA (Fe(III)), with Mn, Zn, KI, H_3_BO_3_ stock (1×), Cu, Co, Mo stock (1×), AAM vitamins stock (1×), and 500 mg L^-1^ of casamino acid, 3 g L^-1^ of KCl, 176.7 mg L^-1^ of L-arginine, 900 mg L^-1^ of L-glutamine, 300 mg L^-1^ of L-aspartic acid, 68.5 g L^-1^ of sucrose and 36.0 g L^-1^ of glucose, along with 20 mg L^-1^ of acetosyringone (pH 5.2).

**N6-AS co-cultivation medium**

N6 stock 1 (1×), N6 stock 2 (1×), N6 stock 3 (1×) N6 stock 4 (1×), N6 stock 5 (1×), N6 stock 6 (1×), 2.0 mg L^-1^ of 2,4-D, 300 mg L^-1^ of casamino acid, 30 g L^-1^ of sucrose, 10 g L^-1^ of glucose (pH 5.2) with 0.5% gellan gum.

**MS-S selection medium**

MS salt ^27^, MS stock 1 (1×), 10 µM of 2,4-D, 78.4 mg L^-1^ of Fe-EDTA, 3 g L^-1^ of casamino acid, 30.0 g L^-1^ of sucrose, 0.8% agar (pH 5.8) with 50 mg L^-1^ of hygromycin and 400 mg L^-1^ of carbenicillin.

**MS-RE regeneration medium**

MS salt ^27^, MS stock 1 (1×), 78.4 mg L^-1^ of Fe-EDTA, 3 g L^-1^ of casamino acid, 50 g L^-1^ of sucrose ^23^, 0.1 µM of 2,4-D, 50 µM of kinetin (pH 5.8) with 0.65% agar, 50 mg L^-1^ hygromycin, and 400 mg L^-1^ carbenicillin.

**MS-RI root induction medium**

Half-strength MS salt ^27^, 30 g L^-1^ sucrose (pH 5.8) with 50 mg L^-1^ of hygromycin and 2 g L^-1^ of Gelrite.

**Supplementary Figures and Tables**


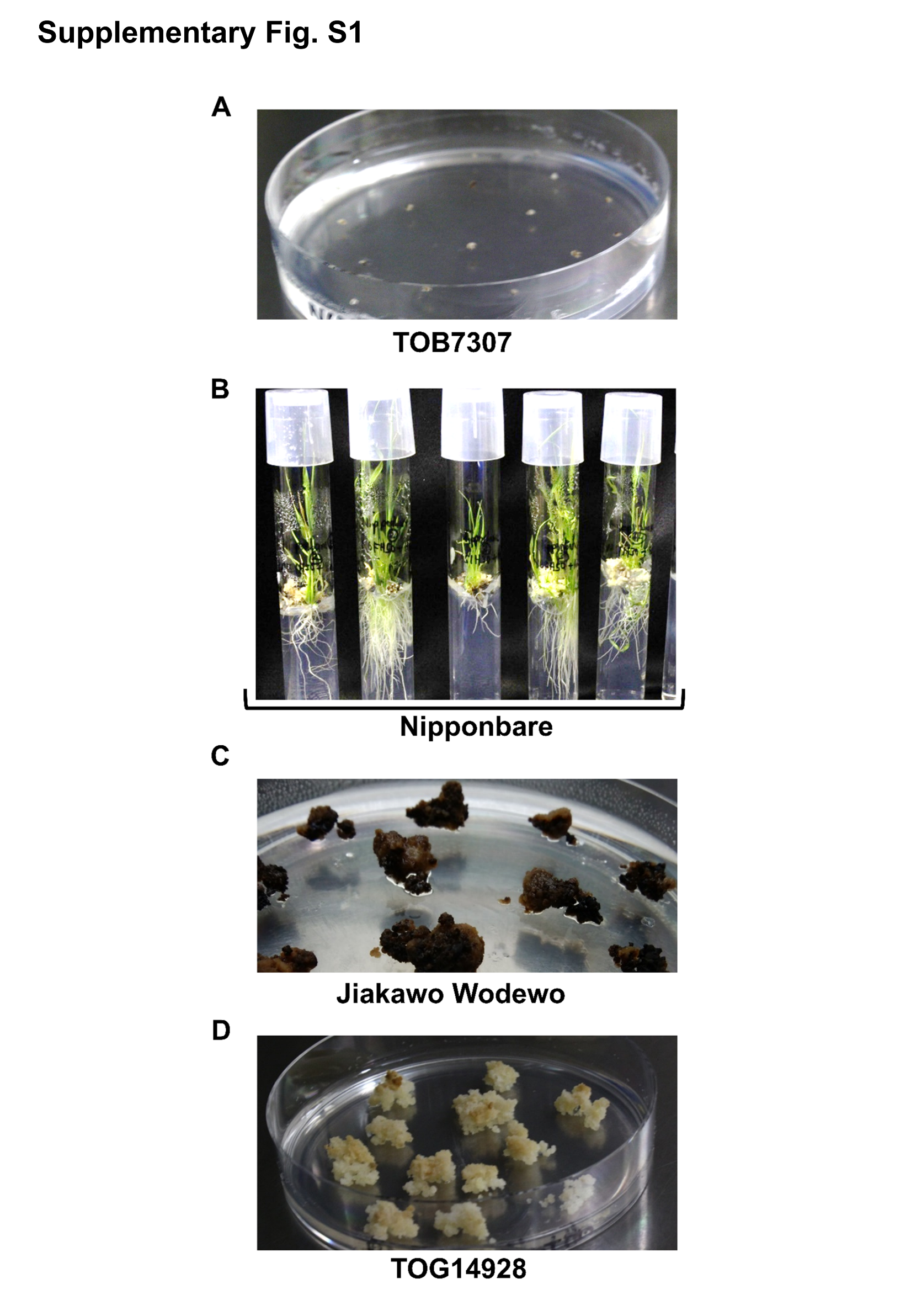


**Figure S1.** Callus induction and the regeneration problem of African rice cultivars. (**A**) Failure to introduce the hygromycin phosphotransferase (*hpt*) gene into an immature embryo of African rice cultivar TOB7307. Immature embryo extraction and transformation was performed as described previously ^42^, and two media conditions for the genotype *(japonica* type 1 and 2 and *indica* type 6, 4, and 5) were tested in this study in both the Nipponbare cultivar and the UVB-super-hypersensitive African rice cultivar TOB7307. (**B**) Regenerated calli from an immature embryo of Nipponbare. (**C**) African rice Jiakawo Wodewo was not selected on the N6D callus induction media due to a failed introduction failure of the hygromycin phosphotransferase (*hpt*) gene. (**D**) Regeneration failure of the African rice cultivar TOG14928.


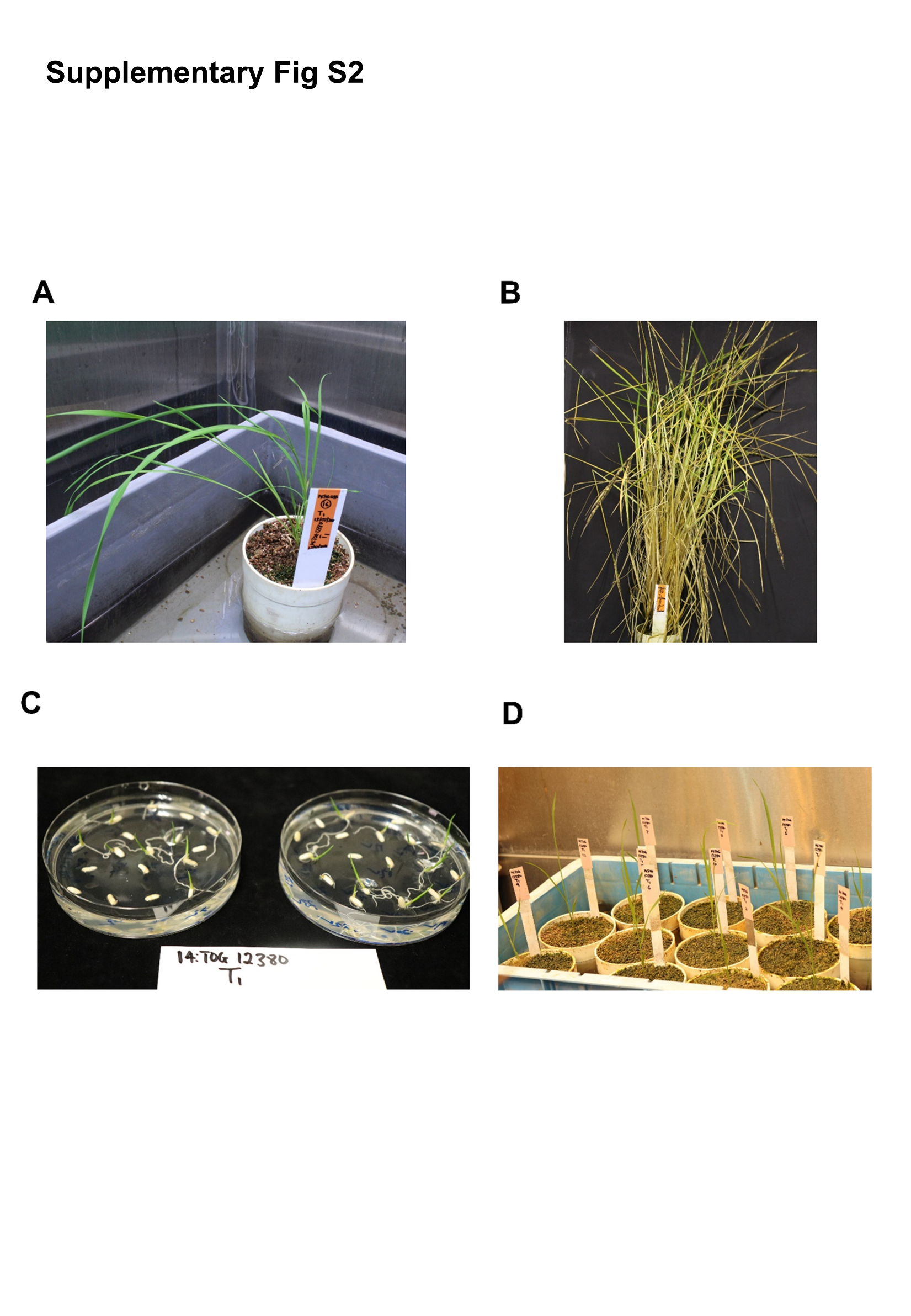


**Figure S2.** Callus induction and regeneration using mature seeds of the African rice plant TOG12380. The procedure for callus induction was performed using a modification of Brisibe et al. (1990) ^23^ by increasing the sucrose concentration to 50 g L^-1^. See Supplementary Materials and Methods for full details of the transformation and regeneration procedures. (**A**) Vegetative phase of TOG12380 T_0_ grown in soil. (**B**) TOG12380 T_0_ at the ripening stage. (**C**) Germination efficiency of the TOG12380 T_1_ seeds. (**D**) Vegetative phase of TOG12380 T_1_ plants grown under visible radiation in a growth chamber.

**Table S1**. **Determination of best condition for callus induction in African rice species**


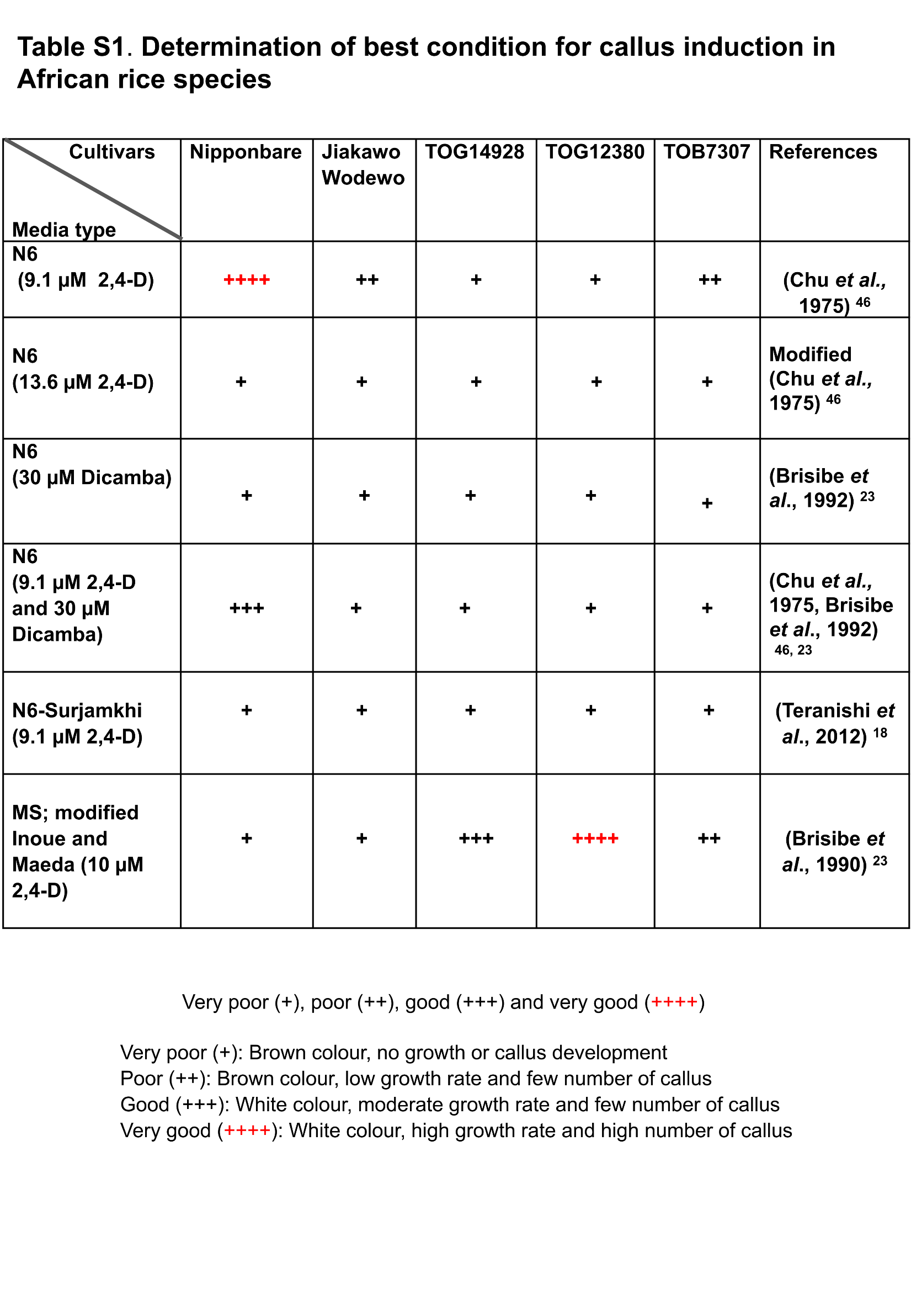


Very poor (+), poor (++), good (+++) and very good (++++)

Very poor (+): Brown colour, no growth or callus development

Poor (++): Brown colour, low growth rate and few number of callus

Good (+++): White colour, moderate growth rate and few number of callus

Very good (++++): White colour, high growth rate and high number of callus


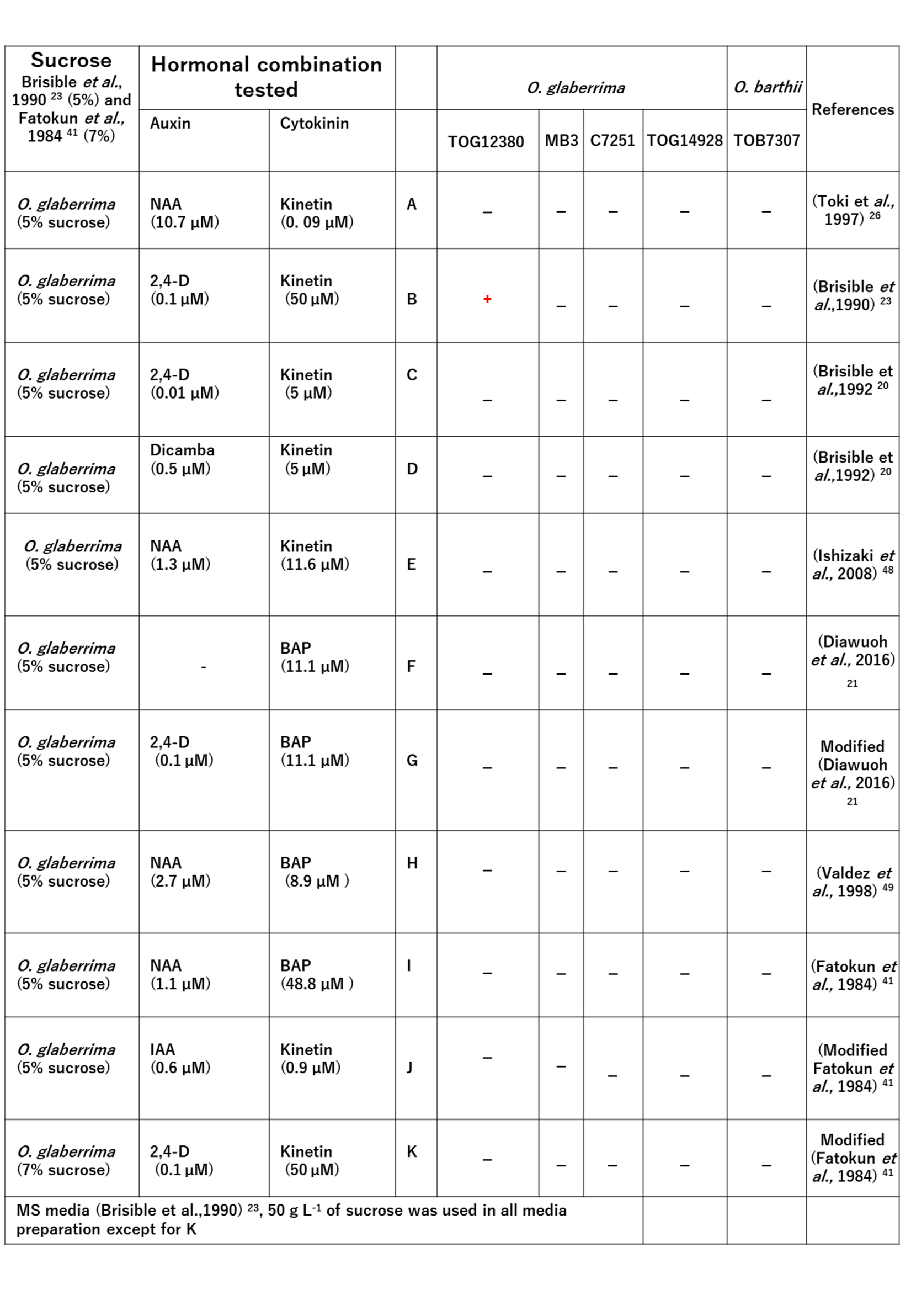
**Table S2. Determination of optimal hormonal concentration for regeneration of *Oryza glaberrima* rice cultivars.**

^_^ = Not regenerated + = Regenerated

**Table S2**. Regeneration was performed by protocol adapted from (Brisible et al., 1990) ^23^ with several modifications of both hormonals (A-J) and sucrose (K). Hormonal combination B was successfully regenerated *O. glaberrima* cultivar TOG12380.

**Table S3. The effects of UVB radiation on tiller number and fresh weight of the above-ground parts of TOG12380-photoreactivation enzyme overexpressing (OxPHR) and parental rice (PL)**


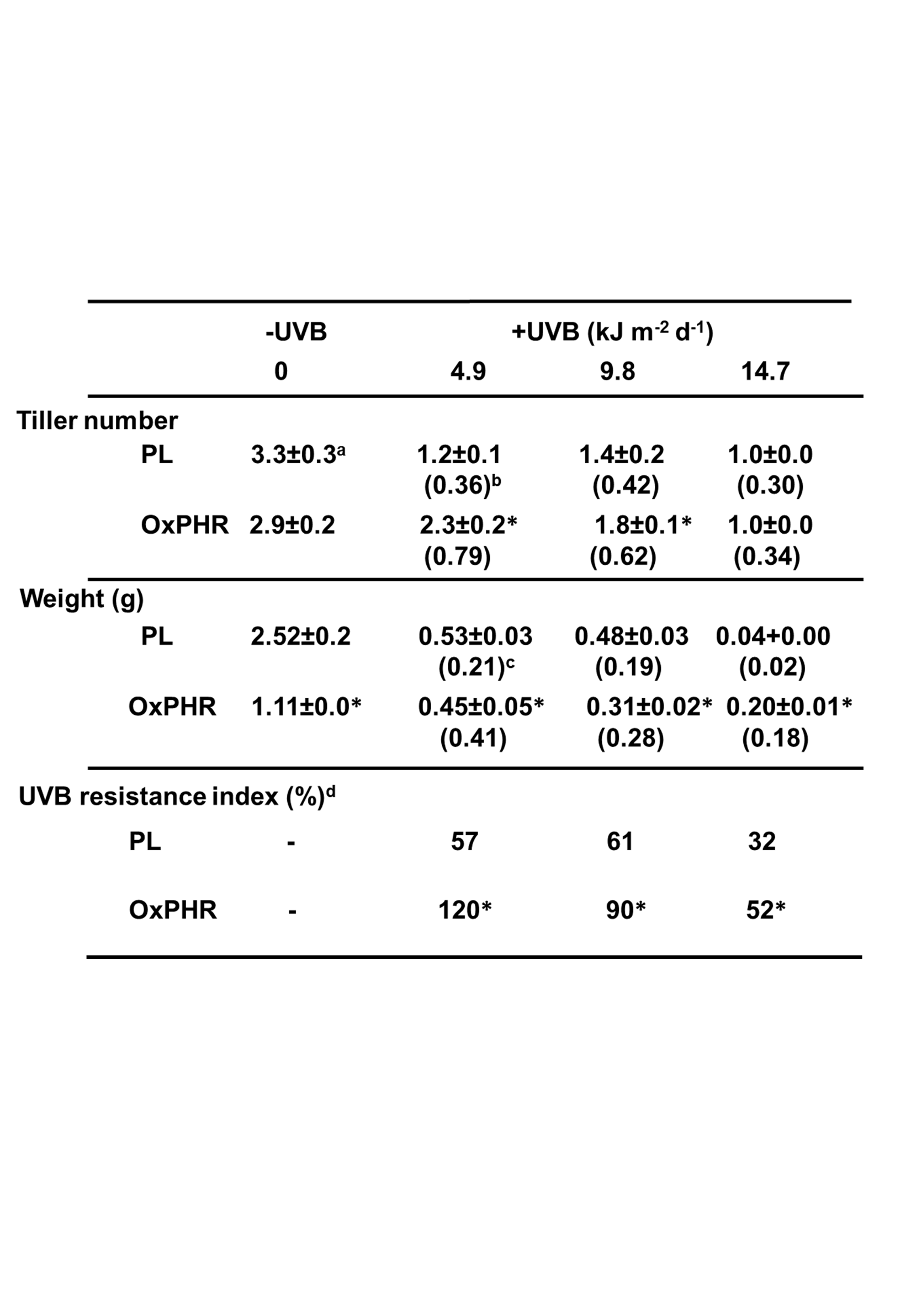


^a^Standard deviation, n=9-10

^b^Ratio of irradiated to unirradiated tiller number = (+UVB)/Control (-UVB)

^c^Ratio of irradiated to unirradiated fresh weight = (+UVB)/Control (-UVB)

^d^Resistance index was determined by summing the value of ratio of irradiated to unirradiated tiller number and fresh weight; Resistance index = (a+b) x100

* Significance differences at P*<0.05*
